# Methicillin-Resistant *Staphylococcus aureus* (MRSA) in Different Food Groups and Drinking Water

**DOI:** 10.3390/foods13172686

**Published:** 2024-08-26

**Authors:** Camino González-Machado, Carlos Alonso-Calleja, Rosa Capita

**Affiliations:** 1 Department of Food Hygiene and Technology, Veterinary Faculty, University of León, E-24071 León, Spain; 2 Institute of Food Science and Technology, University of León, E-24071 León, Spain

**Keywords:** MRSA, fish, eggs, foods of vegetable origin, honey, edible insects, ready-to eat foods, drinking water

## Abstract

Methicillin-resistant *Staphylococcus aureus* (MRSA) has been included by the World Health Organization in its list of “priority pathogens” because of its widespread prevalence and the severity of the infections it causes. The role of food in infections caused by MRSA is unknown, although strains of this microorganism have been detected in various items for human consumption. In order to gain an overview of any possible role of food in MRSA infections, a review was undertaken of studies published between January 2001 and February 2024 relating to MRSA. These comprised research that focused on fish and shellfish, eggs and egg products, foods of vegetable origin, other foodstuffs (e.g., honey or edible insects), and drinking water. In most of these investigations, no prior enrichment was carried out when isolating strains. Three principal methods were used to confirm the presence of MRSA, namely amplification of the *mec*A gene by PCR, amplification of the *mec*A and the *mec*C genes by PCR, and disc diffusion techniques testing susceptibility to cefoxitin (30 μg) and oxacillin (1 μg). The great diversity of methods used for the determination of MRSA in foods and water makes comparison between these research works difficult. The prevalence of MRSA varied according to the food type considered, ranging between 0.0% and 100% (average 11.7 ± 20.3%) for fish and shellfish samples, between 0.0% and 11.0% (average 1.2 ± 3.5%) for egg and egg products, between 0.0% and 20.8% (average 2.5 ± 6.8%) for foods of vegetable origin, between 0.6% and 29.5% (average 28.2 ± 30.3%) for other foodstuffs, and between 0.0% and 36.7% (average 17.0 ± 14.0%) for drinking water.

## 1. Introduction

### 1.1. MRSA

*Staphylococcus aureus* is a bacterium that commonly colonizes the skin, mucous membranes and nasal passages of healthy people and animals. This microorganism is responsible for staphylococcal poisoning, the cause of which is the consumption of foods contaminated with its toxins. Since many people are asymptomatic carriers of *S. aureus*, it may be transmitted to foodstuffs as a result of unhygienic practices on the part of food handlers [1]. Staphylococcal poisoning triggers nausea and vomiting, and it can sometimes also cause diarrhea and abdominal pain [2].

In certain circumstances, *S. aureus* is the etiological agent of infections, generally associated with hospital environments. Methicillin is a semi-synthetic antibiotic widely used for the treatment of staphylococcal infections, although in recent years there has been a marked increase in the prevalence of methicillin-resistant *S. aureus* (MRSA) strains. In addition, these microorganisms can present resistance to a range of antibiotics in clinical use, being colloquially termed “superbugs” [3]. Although in the past MRSA transmission occurred in clinical settings, in recent decades its presence in the community, outside hospital environments, has been described, with MRSA strains having been isolated in foodstuffs and animals used for food around the world [2].

The World Health Organization (WHO) has incorporated MRSA into its list of “priority pathogens” because of its extensive presence and the severity of the infections it triggers, which pose a significant therapeutic challenge [3]. MRSA produces an altered penicillin-binding protein (PBP2a), which confers resistance to all beta-lactam antibiotics. Methicillin resistance in staphylococci is due to the presence of the *mec*A and *mec*C genes, which are found within the staphylococcal chromosome cassette (SCC*mec*), although there are also other genes responsible for methicillin resistance (*mec*B, *mec*D), which have mostly been described in *Macrococcus* spp. [4].

### 1.2. Fish and Seafood

Fish and shellfish have high nutritional value but are often linked to foodborne pathogens. Thus, in many developed countries, such as the United States, European nations, and Canada, these foods are responsible for a considerable percentage of the cases of infection and food poisoning that occur each year [5]. With regard specifically to MRSA, this bacterium has been detected in aquatic animals and fish, even though it is not considered a typical microorganism in their microbiota, and it can contaminate these foods as a consequence of poor hygiene practices, through cross-transfer from surfaces and equipment where it has become present, or as an outcome of contact with infected handlers [6]. Furthermore, MRSA infections are considered a zoonosis, and although there is no clear evidence of direct transmission of MRSA from fish to humans, interaction with the aquatic environment, as well as the handling and consumption of seafood, suggests the possibility that fish contaminated with MRSA might be a source of foodborne infection for humans [7]. It has been demonstrated that MRSA can be transmitted from food handlers to seafood and vice versa [8].

### 1.3. Egg and Egg Products

Eggs are one of the most affordable and nutritious foods eaten by humans. At the time of laying, the inside of the egg is practically sterile, but the shell can become contaminated when passing through the cloaca, or once it is outside the bird. Lack of hygiene during handling, as well as storage and distribution under inadequate conditions, increase shell contamination [9]. Different microorganisms have been detected on eggshells, including *S. aureus*, *Salmonella* spp., *Streptococcus* spp., *Escherichia coli*, *Bacillus* spp. or *Listeria monocytogenes*. Over time, these microorganisms can break through the covering and penetrate the inside of the egg, increasing the risk of foodborne diseases [10]. In the specific case of *Staphylococcus* spp., there are several poultry diseases caused by this microorganism, so eggs are a potential source of infection by antibiotic-resistant *Staphylococcus* strains [11,12].

### 1.4. Foods of Vegetable Origin

Fruit and vegetables are important foods in a healthy diet. In recent years, people’s search for a healthier lifestyle has led to greater consumption of these products [13]. However, the role of such foodstuffs as potential carriers of pathogenic microorganisms has become increasingly clear, with various sources of contamination being recognized, such as dust, soil, manure, irrigation water, or the feces of wild animals. In addition, these products can suffer cross-contamination through contact with storage equipment, or with surfaces in transport vehicles, as well as through direct touching by agricultural workers and food handlers, especially if hygiene practices are poor [14]. Moreover, a range of plant products are often consumed raw, which increases the risk of infection for consumers [4].

The data available for characterizing *S. aureus* have focused principally on clinical settings, foods of animal origin and dairy products, so the information available on fruits and vegetables is very limited [15]. Furthermore, most of the work carried out on the incidence of antibiotic-resistant bacteria has been based on phenotypic studies, with few molecular studies carried out to determine the resistance genes present [16].

### 1.5. Other Foods

The health benefits of honey have attracted the interest of many researchers over the past few decades. This food has important antibacterial properties, with both bacteriostatic and bactericidal effects. The micro-environment of honey is, thus, not favorable for the presence of bacteria, but spore-forming bacteria, such as *Bacillus* and *Clostridium*, can nevertheless be found in it, generally at low levels. The presence of *Staphylococcus* or Enterobacteriaceae is no more than an occasional finding and these microorganisms are probably secondary product contaminants from the air, environment, equipment, or handling. The bacteria isolated from honey seem to be related to the origin of the sample, the quality of the honey, and its state of hygiene [17].

In most developing countries, protein deficiency is a major challenge. It is, therefore, necessary to explore the use of unconventional sources of protein, such as insects. It has been suggested that foods of this nature might be another inadvertent source for the transfer of multidrug-resistant bacteria [18].

Ready-to-eat (RTE) foods can be consumed without any heat treatment or washing, sometimes at the point of sale. Convenience, ease of production, affordability, palatability and availability are some of the attractive factors that make such foods very popular, being enjoyed by people of all classes and age groups, but in particular by workers in urban areas and by young people. In some developing countries, ready-to-eat food vendors have been noted as having limited levels of literacy, leading to a lack of knowledge about good hygiene and food handling practices [19].

RTE products that have frequently been implicated in staphylococcal poisoning include meat and meat products, poultry and egg products, milk and dairy products, salads, bakery goods (particularly cream-filled pastries), and sandwich fillings. However, the foods involved differ from country to country, above all owing to variations in eating habits. Specifically, food contamination by *S. aureus* is due primarily to its ability to enter the food chain through raw materials containing it, improper handling of processed foods, and failure to maintain the cold chain [20].

### 1.6. Water for Human Consumption

Any individual’s survival depends on a range of factors, among them being the availability of water, which constitutes around 60% of the human body. Sources from which water is taken include rivers, lakes, streams, ponds, reservoirs, springs, and wells [21]. Transmission of a number of pathogenic microorganisms present in water can occur through the use of water-related devices, such as showers, fountains, bathtubs, oral irrigators, ice machines, humidifiers, sinks, and toilets. Water sources polluted by antimicrobial-resistant pathogenic bacteria provide unique environments for the spread of antibiotic resistance genes. Consequently, it has been shown that sinks, taps, drains, bathtubs, drinking water fountains, ventilation grilles, and showers may be contaminated with one or more species of multi-resistant bacteria [22].

The presence of *S. aureus* in drinking water has hitherto been little studied, and its possible health risks are unknown. However, there are reports of infections by this microorganism associated with various water sources. Specifically, the presence of MRSA has been detected in water intended for human consumption. In consequence, there is a need to monitor any presence of opportunistic bacteria, such as staphylococci, with control measures appropriate for the protection of human health, this being especially necessary in crowded public places [23].

Two reports have been recently published on the prevalence and types of MRSA in meat and meat products [24] and in dairy products [25]. However, a review on MRSA in other foods and drinking water is lacking. In the light of all the above, the present work was undertaken with the objective of compiling an overview of the literature available on the prevalence of MRSA in a variety of products for human consumption, determining which types are most frequent in given foodstuffs. A description of the methodology used in each of the studies surveyed was also incorporated, making it possible to elucidate which approaches offer better detection, identification, and typing in MRSA-positive samples.

## 2. Materials and Methods

Various databases including Web of Science, Scopus, PubMed, and ScienceDirect were consulted to compile a list of all relevant studies on the prevalence of MRSA in different foods. Studies published between January 2001 and February 2024 were traced. No conditions regarding date, language, article type, or text availability were imposed. A total of 57 articles were selected for fish and shellfish, 18 for eggs and egg products, 18 for vegetables, mushrooms, and fruits, 47 for miscellaneous types of food, such as ready-to-cook or ready-to-eat foods, other prepared items, frozen foods, honey, or insects, and 7 for drinking water.

The details of this research work were drawn up in tables ordered by year of publication and, within the year, in alphabetical order of the authors of the articles. The dates and places of studies, the prevalence of MRSA (in the absence of further clarification, the results shown refer to the detection of the *mec*A gene), and the typing of the MRSA strains found in the study were analyzed. A numerical code explained at the foot of the tables was established to identify the protocols followed in the various research works within the different methodologies for identifying MRSA.

## 3. Results

### 3.1. Fish and Seafood

A total of 57 articles were reviewed for fish and seafood (crustaceans and mollusks), as shown in Table 1. In 19 investigations (33.3% of the total) no prior enrichment was performed for the isolation of MRSA. Only a few articles, six in all (10.5%) described the performance of double enrichment of the samples, and none referred to triple enrichment. The most widely used culture medium was tryptone soy broth (TSB), employed in 16 investigations (28.1%). Six cases (10.5%) were supplemented with 10% NaCl and 1% sodium pyruvate. In seven studies (12.3%), a selective chromogenic medium for MRSA was used, and in five (8.8%), oxacillin resistance screening agar base (ORSAB) supplemented with oxacillin (2 mg/L) was used. Three main methods were used to confirm the presence of MRSA: in the majority of the research, 40 studies (70.2%), there was amplification of the *mec*A gene carried out using a polymerase chain reaction (PCR), while in 7 investigations (12.3%) there was amplification of the *mec*A and the *mec*C genes by PCR, and 15 publications (26.3%) referred to the use of a disc diffusion test for susceptibility to cefoxitin (30 μg) and oxacillin (1 μg). In 34 investigations (59.6%), MRSA strains were detected, the prevalence being less than 3% in the majority of studies with positive samples, although some reports referred to a prevalence as high as 30%.

### 3.2. Eggs and Egg Products

Eighteen articles relating to eggs and egg products were reviewed (Table 2). In eight publications (44.4%) there was no indication of prior enrichment being undertaken. A single publication (5.6%) referred to the carrying out of double enrichment of the samples. Three principal methods for confirming the presence of MRSA were used. In 13 investigations, the majority (72.2%) of the previous studies, amplification of the *mec*A gene was achieved by using PCR. In three articles (16.7%), reference was made to amplification of the *mec*A and *mec*C genes by PCR, and in one single research work (5.6%), a disc diffusion test for susceptibility to cefoxitin (30 μg) and oxacillin (1 μg) was performed.

### 3.3. Foods of Plant Origin and Mushrooms

Eighteen publications covering vegetables, mushrooms, and fruits were reviewed (Table 3). No prior enrichment was carried out in six studies (33.3% of the total). In only one investigation (5.6%) was a double enrichment of the samples undertaken. The most widely used culture medium was TSB, employed in five investigations (27.8%), in three cases (16.7%) supplemented with 10% NaCl. Just one of the studies consulted used a selective chromogenic medium for MRSA. Use was made of three main methods for confirming that MRSA was present. In 15 articles, a strong majority (83.3%), amplification of the *mec*A gene by means of PCR was described. Five publications (27.8%) reported amplification of the *mec*A and *mec*C genes with this same technique, whilst one single article (5.6%) noted use of a disc diffusion test for susceptibility to cefoxitin (30 μg) and oxacillin (1 μg). In 12 of the investigations (66.7%) MRSA was detected, with its prevalence varying.

### 3.4. Other Foods

A total of 47 articles were evaluated for different types of foods, including prepared and cooked foods, RTE, frozen, honey, and insects (Table 4). In a substantial number of the publications, specifically 22 (46.8% of the total), no prior enrichment was reported as having been undertaken. Few works performed double enrichment of the samples (seven; 14.9%). The most frequently used culture medium was TSB, which was employed in ten studies (21.3%); in a good few cases, five in all (10.6%), it was supplemented with 7.5% NaCl. Only one investigation (2.1%) used a selective chromogenic medium for MRSA. The methods utilized to confirm the presence of this bacterium were primarily as follows: 32 publications, a clear majority (68.1%), describe amplification of the *mec*A gene by means of PCR. In seven articles (14.9%), there was reference to amplification of the *mec*A and *mec*C genes by that same method, and five of the articles (10.6%) indicated that confirmation of MRSA was achieved by disc diffusion tests for susceptibility to cefoxitin (30 μg) and oxacillin (1 μg). MRSA was detected in 32 of the articles, representing 68.1% of the total.

### 3.5. Drinking Water

Seven articles relating to MRSA in drinking water were reviewed (Table 5). In three of the articles (nearly half, at 42.9%) it was indicated that no prior enrichment was undertaken. In none of the works was a double enrichment of the samples performed. Only one research work (14.3%) indicated the use of a selective chromogenic medium for MRSA. Three main methods were used to confirm the presence of MRSA: in two articles (28.6%) reference was made to the amplification of the *mec*A gene by PCR, one of the articles (14.3%) described amplification of the *mec*A and *mec*C genes by this same technique, and in two instances (28.6%) there was testing for susceptibility to cefoxitin (30 μg) and oxacillin (1 μg) employing the disc diffusion method.

**Table 1:** Prevalence of MRSA in fish and shellfish (in the absence of clarification, the prevalence is considered based on the detection of *mec*A gene).

Samples	Period	Location	Detection Method	Prevalence	Identification	Reference
136 fish samples from fish markets: 71 rainbow trout and 65 gibel carp		Northern Greece	-Homogenized: 1.3.4. -Two methods: ·1.1. ·1.3.4. -2.1.2 -3.4.	None of the *S. aureus* isolates were resistant to oxacillin and glycopeptides		[26]
1 fish sample	March–July 2008	The Netherlands	-First: 1.4.1. -Second: 1.9.2. -2.9.-2.12.1. -3.2.	None		[27]
165 *S. aureus* strains isolated from different food samples: 12 fishery products	2003–2006	Korea	-1.1. -3.4.-3.2.	4/165 (2.4%): 2 fish samples (rockfish and sea bass)	-ST1 (sea bass) -ST72 (rockfish)	[28]
200 samples of retail RTE raw fish (sashimi) from retail grocery stores of five supermarket chains	January–February 2010	Hiroshima (Japan)	-1.3.2. -2.1.-2.1.3.-2.4.1. -3.2.-3.4.	5/200 (2.5%): 3 MRSA isolates did not carry the *mec*A gene	ST8/t1767 (2 MRSA isolates harboring *mec*A)	[1]
298 fishery products from retail outlets (fresh products, frozen products, salted fish, ready-to-cook products, smoked fish, fish roes, non-frozen surimi and other RTE products)	January 2008–May 2009	Vigo, Galicia (Spain)	-Homogenized 1.2. -1.1. -2.1.1. -3.4.-3.2.	None		[29]
25 aquatic products from markets, grand large hotel and farms	April–November 2011	Anhui (China)	-1.1. -3.4.	None		[30]
54 fishery products from wholesale marts	February–October 2011	Seoul and Gyeonggi, Chungcheong, Gyeongsang, Jeolla, and Gangwon (Korea)	-1.3.3. -2.1.2. -3.4.-3.2.	4/22 (18.2%) of *S. aureus* isolates		[31]
100 samples of RTE fish products (salted pilchards, smoked mackerel, dried chub mackerel, and marinated anchovies) from retail outlets	March–May 2011	Greece	-1.3.5. -2.1.2.-2.4. -3.2.-3.4.	2/100 (2%) from smoked mackerel and marinated anchovies	-ST359/t316 -ST5/t548	[32]
30 fish samples and 18 samples of prepared foods made with fish from the kitchens of public hospitals	July 2011–May 2012	Salvador (Brazil)	-First: 1.4.1. -Second: 1.9.1. -2.11. -3.9.	-9/30 (30%) of raw fish samples -4/18 (22.2%) of the prepared foods with fish		[33]
330 imported fresh fish samples: 50 red mullet, 30 red sea bream, and 30 emperor fish from each country (Egypt, India, and Yemen)		Jordan	-1.3.1. -2.1.1. -3.4.-3.2.	21/156 (13.5%) of *S. aureus* isolates: 13 from Egypt, 8 from Yemen, and none from India		[34]
200 food samples sold in a black market at an EU border: fish and fish products (31%)	July 2012–February 2013	Galati (Romania)	-1.1. -3.4.-3.3.	None		[35]
3 aquatic products from different food markets	August–October 2010	Wuhou District of Chengdu City, Sichuan Province (China)	-1.5. -2.1.1. -3.2.-3.4.	None		[36]
105 marine fish samples from retail fishery outlets		Ernakulum, Alappuzha, and Kottayam (India)	-Homogenized: 1.5.1. -1.1. -2.1.-2.10. -3.4.-3.2.	1/252 (0.4%) of *S. aureus* isolates		[37]
600 samples (fresh and frozen, farm and marine): 150 marine shrimps, 150 farmed shrimps, 150 farmed fish, and 150 marine fish	September 2013–September 2014	Tehran (Iran)	-1.6. -2.1.1. -3.4.-3.2.	49/206 (23.8%) of *S. aureus* isolates: 3 (16.7%) fresh marine shrimp samples, 5 (16.7%) frozen marine shrimp samples, 7 (46.6%) fresh farm shrimp samples, 8 (38%) frozen farm shrimp samples, 6 (16.2%) fresh marine fish samples, 7 (13.2%) frozen marine fish samples, 5 (41.7%) fresh farm fish samples, and 8 (40%) frozen farm fish samples		[38]
44 samples from retail shops: 11 salmon, 12 pangasius (shark catfish), 11 shrimp, and 10 oysters	August–October 2014	Berne (Switzerland)	-Two methods: ·1.1. and 2.5. ·1.7. and 2.5. -3.7.-3.4.	None		[39]
200 samples of raw and RTE food illegally sold from a market at the eastern EU Border: 61 fish and fish products (smoked or canned fish), from the Republic of Moldova, Ukraine, and Bulgaria	July 2012– February 2013	Galati (Romania)	-1.1. -2.1.2. -3.10.	0.01% fish canned in oil with herbs and smoked fish	*spa* type t1606	[40]
35 seafood samples from retail markets and landing centers: fish products, salt, seawater and surface swabs		Mumbai (India)	-1.1. -2.1.1. -3.4.-3.2.	1/35 (2.86%): isolated from the salt		[41]
233 samples from markets and aquaculture farms: 137 finfishes, 31 crustaceans, 26 mollusks, and 39 environmental samples	July 2012–April 2015	Ernakulam, Kottayam, and Alappuzha (India)	-1.3.1. -2.1.5.-2.2.-2.6. -3.4.-3.2.	31/233 (13.4%): 13.8% finfish, 9.3% crustaceans, 12% mollusks and 15.3% in the environment		[42]
RTE 149 sushi and 51 sashimi from different food outlets	August–December 2014	Klang Valley (Malaysia)	-First: 1.2. -Second: 1.3.4. -2.1.1. -3.4.	4/52 (7.7%) resistant to cefoxitin: 3 sushi and 1 sashimi		[43]
9 CPS isolates recovered from raw fish samples		Turkey	-Homogenized: 1.7. -1.1. -2.1. -3.5.-3.2.	9/9 (100%)		[44]
1552 seafood samples from retail outlets and the fish processing industry: 379 chilled whole cleaned fish, 308 fresh fish, 614 frozen whole cleaned fish, 124 samples of processed fish and prawn, and 76 water and 51 ice samples	2011–2015	Gujarat (India)	-2.1.2.-2.2.	88/1552 (5.67%) were found to be positive for MRSA on MRSA II selective plates: 14 CA-MRSA samples (7 prawn samples, 5 water, and 2 ice)		[45]
40 freshwater fish (*Oncorhynchus mykiss*) and 40 seawater fish (*Sparus aurata*) samples from bazaars and supermarkets	October 2011–December 2012	Bolu (Turkey)	-1.3.4. -2.1.1. -3.2.-3.4.-3.6.	None		[46]
9 raw fish from the big hospitals	June 2015–June 2016	Isfahan (Iran)	-First: 1.2. -Second: 1.3.1. -2.1.1. -3.1.-3.2.	None		[47]
25 fish products from food processing industries	2013–2016	Karachi (Pakistan)	-2.1.1.-2.4.-2.3.-2.5. -3.2.	4/25 (16%)	SCC*mec*A IV/*agr* type II	[48]
Sashimi samples from 20 Japanese restaurants: 21 salmon, 18 tuna, 12 sea bass and 9 others	August–October 2013	Portugal	-1.1. -2.1.4. -3.4.	*Staphylococcus* spp. isolates showed resistance to oxacillin (13.9%) and cefoxitin (7.7%)		[49]
17 seafood and fishery environment samples: 10 samples from a landing center and 7 samples from retail fish markets (sampling plan was repeated within a week to validate the procedure with same sample number)		Kerala (India)	-1.3.1. -2.1.5.-2.2.-2.4.-2.6.-2.10. -3.5.-3.2.	-First sampling: 6/17 (35.2%): ice, *Scylla serrata*, water and *Channa striatus* (at the landing center), and black clam meat (*Villorita cyprinoides*) and Indian anchovy (*Stolephorus indicus*) (retail market) -Second sampling: 4/17 (23.5%): water and ice (at the landing center) and clam meat and ice (retail market)	-First sampling: ·At the landing center: ice (t311), *Scylla serrata* (t15669/t311), water (t15669), and *Channa striatus* (t15669) ·Retail market: black clam meat (*Villorita cyprinoides*) (t121/t311) and Indian anchovy (*Stolephorus indicus*) (t186) -Second sampling: ·At the landing center: water (t15669) and ice (t311/t15669) ·Retail market: clam meat (t15669) and ice (t311/t127)	[50]
267 samples of raw seafood (finfish and shellfish) from retail markets and a fish landing center, and from the aquatic environments (ice, water, and mud)		Kerala (India)	-1.3.1. -2.1.5.-2.2.-2.6.-2.10. -3.2.	65 MRSA isolates	-ST5 (CC5)/*spa* complex I/*spa* type t002: 2 isolates, 1 sample; needle fish (*Xenentodon cancila*) (5%) -ST5 (CC5)/I/t311: 19 isolates, 7 samples; Japanese butter fish (*Psenopsis anomala*), croaker (*Johnius malabaricus*), ice, mud crab (*Scylla serrata*), 2 black clam meat (*Villorita cyprinoides*), and dried Indian prawn (*Penaeus indicus*) (38.8%) -ST772 (CC1)/I/t657: 3 isolates, 2 samples; milk fish (*Chanos chanos*) and water (11.1%) -ST8 (CC8)/II/t121: 5 isolates, 1 sample; black clam meat (5%) -ST5 (CC5)/II/t334: 2 isolates, 1 sample; mackerel (*Rastrelliger kanagurta*) (5%) -ST88 (CC88)/III/t186: 6 isolates, 1 sample; anchovy (*Stolephorus indicus*) (5%) -ST1 (CC1)/III/t127: 1 isolate, 1 sample; ice (5%) -ST6 (CC5)/IV/t711: 4 isolates, 2 samples; mackerel, and flat fish (*Cynoglossus malabaricus*) (11.1%) -New profile (420-256-236-66-82-411-477) and CC/*spa* complex V/t15669: 23 isolates, 6 samples; freshwater prawn (*Macrobrachium rosenbergii*), snake head fish (*Channa striatus*), black clam meat, 2 ice, and 1 water from the landing center (33.3%)	[51]
70 raw fish from restaurants	February 2015–February 2016	Isfahan (Iran)	-1.2. -2.1.1. -3.2.-3.4.	None		[52]
6 fish and fish products confiscated from passengers on flights from 45 non-European Union countries by the Border Authorities	August 2012–July 2015	Bilbao International Airport (Spain) and Vienna International Airport (Austria)	-1.1. -2.1. -3.4.-3.3.	None		[53]
320 raw aquatic food samples from retail outlets and commercial hypermarkets: 142 freshwater fish, 113 saltwater fish, and 65 shrimp	June 2015–June 2016	China	-1.3.3. -2.5. -3.4.-3.5.-3.3.	-10 isolates by cefoxitin disk diffusion tests -9 isolates were *mec*A-positive	-ST1/SCC*mec* III (freshwater fish) -ST25/IVa (freshwater fish) -ST398/III (freshwater fish) -ST59/IVa (freshwater fish) -ST338/IVa (saltwater fish)	[54]
16 ABF and 30 conventional fish products from retail stores	January–December 2012	Iowa (USA)	-1.10.1. -2.1.1.-2.2. -3.2.-3.4.	None		[55]
150 catfish (*Clarias gariepinus*) were collected from Bahr Elbaker canal: swab samples from internal organs (pancreas, liver, kidney, and intestine), gills, and skin		Sharkia Governorate (Egypt)	-1.1. -2.4.-2.1.-2.3.1. -3.4.-3.2.	15/70 (21.4%) of *S. aureus* isolates		[56]
120 dusky kob fish samples (*Argyrosomus japonicus*) from two aquaculture dusky kob farms and Kariega estuary (100 from fish farms and 20 from the wild)	November 2014–October 2015	Cape (South Africa)	-1.3. -2.4. -3.2.-2.10.-3.5.-2.8.	33/202 (16.3%) of *S. aureus* isolates: cultured fish (32/33, 97%) and wild catch (1/33, 3%)	-SCC*mec* I (1), II (1), III (17) (HA-MRSA) -SCC*mec* IVc (10) (CA-MRSA) -Four strains could not be typed	[7,57]
93 *S. aureus* isolates from different food samples in supermarket, food market and retail stores	2014	Chengdu (China)	-3.4.-3.2.	None detected with the *mec*A gene -14/93 (15.05%) resistant to oxacillin: 2 aquatic products		[58]
80 samples of raw shellfish from retail markets: 40 samples of mussels and 40 samples of cockles	January–May 2017	Bangi, Kajang, and Serdang (Malaysia)	-1.3.5. -3.8.-3.2.-3.4.	7/80 (8.75%): green mussel (4/40, 10%) and blood cockle (3/40, 7.5%)		[59]
5 raw processed fish samples from retail vendors		Dhaka (Bangladesh)	-1.1. -2.1. -3.4.-3.2.	1/5 (20%): fish finger	ST361/t315	[60]
80 samples of Maki salmon and Nigiri salmon from 20 sushi outlets	November–December 2017	Copenhagen (Denmark)	-Two methods: ·1.1. and 2.8.-2.7. · 1.3.5. and 2.8.-2.7. -3.1.-3.2.-3.4.-3.7.	None		[61]
19 seafood samples from markets		Tripoli, Regdalin, Sabha, Benghazi, Janzour, and Tobruk (Libya)	-1.1. -2.1.1. -3.4.	None		[62]
8 raw fish from street food vendors	September 2015–May 2016	Bareilly (India)	-2.13. -3.2.-3.1.	None		[63]
511 *S. aureus* isolates of aquatic products (freshwater fish, shrimp, and seafood) from 860 samples	July 2011–June 2016	China	-3.5.-3.3.-3.4.	26/860 (3%) -31/511 (6.07%) of *S. aureus* isolates	-ST59 (15), ST1 (4), ST7 (1), ST338 (4), ST630 (1), ST188 (1), ST3304 (1), ST6 (1), ST45 (1), ST88 (1), and not detected (1) -t437 (18), t127 (4), t091 (1), t034 (1), t002 (1), t085 (1), t114 (1), t189 (1), t116 (1), t1764 (1) and t5554 (1) -SCC*mec* III (5), IVa (21), V (2) and not detected (3)	[64]
104 fish samples from different retail meat shops		Silchar, Assam (India)	-1.7. -2.4.-2.1. -3.1.-3.4.	38/104 (36.53%)		[65]
RTE sashimi from Japanese restaurants in Salvador, Brazil. A total of 127 sashimi samples were collected directly from the takeout service in 16 restaurants			-3.1.-3.2.	*S. aureus* was found in 73% of the sashimi samples, including sashimi from tuna (75.5% of samples) and salmon (72.5% of samples). Among those positive samples, 37% were contaminated with MRSA strains, which were found among 38.8% of salmon sashimi and 34.0% of tuna sashimi		[66]
53 samples (dried, fried, and stewed) of the RTE shellfish (*Corbiculid heterodont*) from markets		Yenagoa, Bayelsa State (Nigeria)	-2.4. -3.1.-3.4.-3.2.	35/65 (53.8%) of *S. aureus* isolates -50% of MRSA isolates showed positive for the *mec*A gene		[67]
959 samples representing 8 types of animal-based foods (pork, chicken, beef, duck, lamb, aquatic products, eggs, and milk) were collected from randomly selected local markets (including 21 supermarkets and 18 wet markets)	July 2018–August 2019	Shanghai (China)	-1.8. -2.1.1. -3.4.-3.2.	None		[68]
104 fish samples from retail meat shops	January–April 2017	Silchar and Imphal (Northeast India)	-1.7. -2.4.-2.1. -3.1.-3.2.	38/104 (36.53%): 10.34% isolates harbored the *mec*A gene		[69]
50 samples of salted fish from markets: 25 moloha and 25 fesikh		Kafrelsheikh (Egypt)	-1.1. -2.1.-2.1.1. -3.4.-3.2.	26/50 (52%): 8 moloha and 12 fesikh		[70]
60 salted fish samples from random places: salted sardine, Feseikh, and Molouha (20 each)		Kafrelsheikh Governorate (Egypt)	-1.1. -2.1. -3.2.	12/60 (20%): 6 salted sardine, 4 Feseikh, and 2 Molouha		[71]
44 fish and seafood samples from various retail outlets: 13 fish, 7 mussels and clams, 15 crustaceans (shrimp), and 9 cephalopods (squid and octopus)	Spring of 2019	Gdansk, Gdynia, and Sopot (Poland)	-1.12. -2.2.	26/44 (59%): 6 cephalopods, 10 crustaceans, 4 mollusks, and 6 fish		[5]
10 frozen fish meat samples from 5 open markets		Benin City (Nigeria)	-1.1. -2.4.-2.15. -3.4.	None		[72]
44 samples of fish	January–June 2017	Southern Assam (India)	-1.7. -2.4. -3.1.	13/73 (17.8%) of *S. aureus* isolates resistant to both oxacillin and cefoxitin antibiotics (41.93% fish samples)		[73]
50 freshly preserved salted fish		Gwadar Balochistan area (Pakistan)	-1.7. -2.4. -3.4.-3.2.	13/50 (26%)		[74]
173 samples comprising diverse piscine fauna from retail markets	August 2019–February 2020	Assam (India)	-1.3.1. -2.4. -3.4.-3.8.-3.2.	23/95 (24.21%) of *S. aureus* isolates	ST88 (23): -SCC*mec* Type IV/*agr* I (4): t2526 (3) and not assigned (1) -SCC*mec* not assigned/*agr* III (18): t2526 (17) and not assigned (1) -SCC*mec* Not assigned/*agr* I/t2526 (1)	[75]
180 fish samples from fish markets: finfishes, crustaceans, and mollusks (60 of each)	June 2021 –June 2022	Wayanad district, Kerala (India)	-1.3. -2.1.6. -3.1.-3.2.	-Finfish: 1/54 (1.80%) of *S. aureus* isolates (most of the isolates were resistant to oxacillin (11.11%), followed by cefoxitin (7.4%)) -Crustaceans: 4/25 (16%) of *S. aureus* isolates (most of the isolates showed resistance to oxacillin (24%), followed by cefoxitin (16%)) -Mollusks: none (from a total of 43 *S. aureus* isolates, most of the isolates showed resistance to oxacillin and none of them revealed resistance to cefoxitin)		[76]
20 *S. aureus* isolates from various public bazaars and supermarkets: 11 seawater fish (*Sparus aurata*), 8 freshwater fish (*Oncorhynchus mykiss*), and 1 seawater fish (*Dicentrarchus labrax*)		Bolu (Northwest Turkey)	-3.2.-3.4.	None		[77]
498 seafood samples from the fish market and the fish processing industries: 108 fresh (raw), 79 chilled, 64 frozen, and 124 processed fish samples, and 76 water and 47 ice samples	2012–2017	Veraval region, Gujarat (India)	-ISO 6888–1 and ISO 6888–2:2003 -2.1.-2.25.-2.2. -3.2.-3.4.	15/498 (3%): 3 fresh, 4 chilled, 2 frozen, and 6 processed fish	MRSA strain isolated from dried ribbon fish: ST 243/*spa* type t021/SCC*mec* type V	[8]
65 fish samples from different foodstuffs	August–November 2021	Kafr El-Sheikh Governorate (Egypt)	-2.6. -3.3.	6/65 (9.2%) (none was positive for *mec*C)		[78]
240 samples of *Mugil cephalus* were chosen from various fish retail marketplaces: mullet fish skin and dorsal muscle samples (120 each) were collected	May 2022–March 2023	Sharkia Governorate (Egypt)	-1.2. -2.1.1.-2.6. -3.2.-3.4.	5/45 (11.1%) of *S. aureus* isolates: 4 skin and 1 muscle		[6]

**Meaning of the numbers used in the detection method column. 1. Pre-enrichment.**: 1.1. Without pre-enrichment; 1.2. Pre-enrichment in buffered peptone water, BPW (1.2.1. BPW containing 5% NaCl); 1.3. Pre-enrichment in tryptone soy broth, TSB [1.3.1. TSB containing 10% NaCl and 1% sodium pyruvate; 1.3.2. 2× TSB containing 10% NaCl and 1% sodium pyruvate; 1.3.3. TSB containing 10% NaCl; 1.3.4. TSB containing 7.5% NaCl; 1.3.5. 2 × TSB containing 10% defibrinated sheep’s blood; 1.3.6. TSB supplemented with 75 mg/L aztreonam and 4 mg/L cefoxitin; 1.3.7. TSB supplemented with 75 mg/L aztreonam and 3.5 mg/L cefoxitin; 1.3.8. TSB supplemented with 50 mg/L aztreonam and 3.5 mg/L cefoxitin; 1.3.9. 2× TSB containing 15% NaCl; 1.3.10. TSB containing 6.5% NaCl; 1.3.11. 2× TSB-YE (yeast extract); 1.3.12. TSB supplemented with 70 mg/mL NaCl; 1.3.13. TSB supplemented with 5 mg/L oxacillin; 1.3.14. TSB supplemented with 7.5 mg/L aztreonam and 5 mg/L cefoxitin; 1.3.15. TSB containing 7% NaCl; 1.3.16. TSB containing 6.5% NaCl and 0.3% yeast extract; 1.3.17. TSB containing 7% NaCl, oxacillin (2 μg/mL) and polymyxin B (5 μg/mL); 1.3.18. TSB containing 2.5% NaCl, aztreonam (20 mg/L) and cefoxitin (3.5 mg/L); 1.3.19. TSB containing cefoxitin (3.5 mg/L); 1.3.20. TSB containing 0.6% yeast extract; 1.3.21. TSB containing 4% NaCl, 1% mannitol, 4 mg/L cefoxitin, and 75 mg/L aztreonam; 1.3.22. TSB supplemented with 75 mg/L aztreonam and 3.5 mg/L cefataxime]; 1.4. Mueller–Hinton Broth, MHB [1.4.1. MHB containing 6.5% NaCl and methicillin (4 mg/L); 1.4.2. MHB containing aztreonam (100 mg/L), methicillin (4 mg/L), NaCl (4.0%), NaN3 (100 mg/L), and polymyxin B (10 mg/L); 1.4.3. MHB containing 6.5% NaCl; 1.4.4. MHB containing 7.5% NaCl; 1.4.5. MHB containing 6% NaCl]; 1.5. Brain heart infusion broth, BHI (1.5.1. BHI containing 6.5% NaCl; 1.5.2. BHI containing 10% NaCl; 1.5.3. BHI containing 2% NaCl; 1.5.4. BHI containing 7% NaCl); 1.6. Giolitti–Cantoni broth, GCB (*S. aureus* selective enrichment medium) containing 0.1% potassium tellurite; 1.7. Peptone water, PW; 1.8. Sterile Butterfield’s phosphate-buffered dilution water (BPD); 1.9. Saline solution; 1.10. Phosphate buffered saline (PBS); 1.11. Baird– Parker broth, BP (1.11.1. 2× BP broth; 1.11.2. 2× BP broth with tellurite enrichment); 1.12. Giolitti–Cantoni broth, GCB (1.12.1. GCB supplemented with 3.5% potassium tellurite solution); 1.13. Peptone broth; 1.14. Thioglycolate bouillon; 1.15. Mannitol salt broth, MSB; 1.16. Phenol red mannitol broth, PHMB [1.16.1. PHMB containing ceftizoxime (5 μg/mL) and aztreonam (7.5 μg/mL); 1.16.2. PHMB containing ceftizoxime (5 μg/mL) and aztreonam (75 μg/mL); 1.16.3. PHMB containing ceftizoxime (0.4 μg/mL) and aztreonam (75 μg/mL); 1.16.4. PHMB containing cefoxitin (5 mg/L) and aztreonam (75 mg/L); 1.16.5. PHMB containing oxacillin (4 mg/L); 1.16.6. PHMB containing 7% NaCl; 1.16.7. PHMB containing oxacillin (5 mg/L) and aztreonam (75 mg/L); 1.16.8. PHMB containing oxacillin (4 mg/L) and aztreonam (75 mg/L); 1.16.9. PHMB containing ceftizoxime (4 μg/mL) and aztreonam (75 μg/mL)]; 1.17. Enrichment broth containing 10 g/l tryptone, 75 g/l NaCl, 10 g/l mannitol and 2.5 g/l of yeast extract (0.25% yeast extract, 1% mannitol, 1% tryptone, and 7.5% NaCl); 1.18. Enrichment broth containing 7% salt; 1.19. Contrast TM MRSA broth; 1.20. Cooked meat broth; 1.21. Nutrient broth containing 7.5% NaCl and oxacillin (6 μg/mL); 1.22. MRSA enrichment broth; 1.23. Sodium chloride broth 10%; 1.24. Nutrient broth. **2. Solid media**: 2.1. Baird–Parker agar, BP [2.1.1. BP supplemented with egg yolk tellurite emulsion; 2.1.2. BP supplemented with rabbit plasma fibrinogen (BP-RPF); 2.1.3. BP with cefoxitin (4 µg/mL); 2.1.4. BP selective media; 2.1.5. BP supplemented with antibiotics; 2.1.6. BP supplemented with egg yolk and potassium tellurite; 2.1.7. BP-RPF containing cefoxitin (3.5 µg/mL) and aztreonam (75 µg/mL) (*Staphylococcus*-selective agar); 2.1.8. BP supplemented with egg yolk tellurite emulsion and sulfamethazine]; 2.2. Selective MRSA agar (BBL CHROM agar MRSA; BD)/MRSA chromogenic agar; 2.3. Blood agar, BA (2.3.1. BA supplemented with sheep’’s blood; 2.3.2. 5% horse blood agar and a 10 μg cefoxitin disk; 2.3.3. 5% bovine blood agar); 2.4. Mannitol salt agar, MSA (2.4.1. MSA with 2 µg/mL oxacillin; 2.4.2. MSA with 4 µg/mL cefoxitin; 2.4.3. MSA supplemented with 75 mg/L aztreonam and 6 mg/L oxacillin; 2.4.4. Mannitol salt phenol red agar, MSPRA; 2.5*. S. aureus*-selective CHROM agar plates; 2.6. Oxacillin-resistance-screening-agar-base (ORSAB) supplemented with oxacillin (2 mg/L); 2.7. MacConkey agar; 2.8. Chocolate agar; 2.9. CNA plates (Columbia colistin and nalidixic acid); 2.10. Oxacillin Salt Screen Agar^®^ (MHA with 4% NaCl and 6 µg/mL oxacillin)/oxacillin agar screen sensitivity disk, agar-N supplemented); 2.11. MRSA-ID; 2.12. Columbia agar (CA) (2.12.1. CA supplemented with 5% sheep’s blood); 2.13. MRSA screening; 2.14. Brilliance MRSA Agar (chromogenic selective agar for MRSA); 2.15. Oxoid oxacillin resistance screening agar, ORSA; 2.16. MRSA select^®^ (BioRad) agar (selective chromogenic MRSA agar); 2.17. Hypertonic mannitol agar (HMA); 2.18. MRSA select medium; 2.19. Chrom-ID MRSA agar; 2.20. SA select agar; 2.21. chromogenic media (MRSA-Ident-Agar); 2.22. Tryptone soy agar, TSA (2.22.1. TSA with 5% sheep’s blood and 0.1% esculin; 2.22.2. TSA with 5% sheep’s blood); 2.23. OXA agar oxoid; 2.24. Spectra MRSA (Remel Diagnostics, Lenexa, KS); 2.25. HiChrome MeReSa agar plates with cefoxitin and methicillin supplement (HiMedia). **3. Identification of MRSA**: 3.1. Cefoxitin (30 μg) and oxacillin (1 μg) disk diffusion susceptibility tests; 3.2. PCR-based amplification of *mec*A gene; 3.3. PCR-based amplification of *mec*A and *mec*C genes; 3.4. Antimicrobial susceptibility testing; 3.5. Cefoxitin disk diffusion method; 3.6. Determination of beta-lactamase production; 3.7. Susceptibility to cefoxitin and doxycycline using disk diffusion according to CLSI; 3.8. Oxacillin agar plate; 3.9. MRSA latex agglutination test (penicillin-binding protein 2a latex agglutination test); 3.10*. S. aureus spa* typing; 3.11. Detection of MRSA by the MPN-PCR method; 3.12. Oxacillin E-test strips; 3.13. MICs of oxacillin by broth microdilution method; 3.14. Oxacillin disk diffusion method; 3.15. Micronaut-s MRSA/IFSG GP 4 (Merlin Diagnostika, Bornheim, Germany); 3.16. SPOT Staphytect Plus (Oxoid,DR0100M), which is a latex agglutination test for the detection of aggregation factor, protein A, and some polysaccharides found in MRSA isolates; 3.17. MALDI-TOF-MS (mass spectrometry); 3.18. StaphType DNA microarray assay. Baird–Parker: BP; brain heart infusion: BHI; buffered peptone water: BPW; coagulase-positive *Staphylococcus*: CPS; Giolitti–Cantoni broth: GCB; mannitol salt agar: MSA; mannitol salt broth: MSB; Mueller–Hinton agar: MHA; Mueller–Hinton broth: MHB; not typable: NT; peptone water: PW; phenol red mannitol broth: PHMB; ready-to-eat: RTE; tryptone soya agar: TSA; tryptone soya broth: TSB.

**Table 2:** Prevalence of MRSA in egg and egg products (in the absence of clarification, the prevalence is considered based on the detection of the *mec*A gene).

Samples	Period	Location	Detection Method	Prevalence	Identification	Reference
270 raw egg samples	July 2008–March 2014	China	-First: 1.2. -Second: 1.3. -2.1.1. -3.4.-3.2.	None		[79]
3 eggs confiscated from passengers on flights from non-EU countries	April 2012–June 2013	International Bilbao Airport (Spain)	-1.1. -3.4.-3.3.	None		[80]
2 egg products from different food markets	August–October 2010	Wuhou District of Chengdu City, Sichuan Province (China)	-1.4. -2.1.1. -3.2.-3.4.	None		[36]
455 samples of Indian Rojak food items were collected from 35 different Indian Rojak stalls: 321 cooked items with or without re-heating, 99 serving portions of raw vegetables, and 35 serving portions of gravy	September 2011–January 2012	Singapore	-1.1. -2.1. -3.2.-3.6.-3.5.	1/11 of *S. aureus* isolates (9.09%): 1 egg		[81]
227 *S. aureus* isolates from RTE food and food contact surfaces at retail	2011–2014	Singapore	-3.2.-3.6.-3.5.	1/227 (0.44%) of *S. aureus* isolates: 1 fried egg	ST80/t1198/SCC*mec* IV (CA-MRSA)	[82]
150 samples of chicken products from different farms and markets: 30 unhatched eggs and 30 table eggs		Luxor (Egypt)	-Inoculated: 1.2. -1.1. -2.4. -3.2.	14/21 (66.7%) of *S. aureus* isolates		[83]
7 eggs confiscated from passengers on flights from 45 non-European Union countries by the Border Authorities	August 2012–July 2015	Bilbao International Airport (Spain) and Vienna International Airport (Austria)	-1.1. -2.1. -3.4.-3.3.	None		[53]
2500 eggs and egg products from supermarket outlets	2015	China	-1.7. -2.1.-2.2. -3.2.-3.4.	14/43 (32.6%) of *S. aureus* isolates of eggs and egg products		[84]
93 *S. aureus* isolates from different food samples in supermarkets, food markets, and retail stores	2014	Chengdu (China)	-3.4.-3.2.	None detected with the *mec*A gene -14/93 (15.05%) resistant to oxacillin: 1 egg product		[58]
300 eggs (220 chicken eggs and 80 quail eggs) from different retail shops and farms	January–June 2017	Mymensingh (Bangladesh)	-1.6. -2.4. -3.2.-3.4.	3/23 (13.04%) of *S. aureus* isolates: chicken eggs -*S. aureus* isolates resistant to oxacillin: quail eggs (1/4, 25%) and chicken eggs (6/23, 26.1%)		[11]
300 table eggs from retail shops	December 2015–May 2016	Haripur (Pakistan)	-1.1. -2.4. -3.2.-3.4.	33/300 (11%)	-ST772 (31/33, 94%)/SCC*mec* IV: t657 (30) and t8645 (1) (CA-MRSA) -ST8/IV/t8645 (2/33, 6%) (associated with USA300)	[12]
290 eggs comprising 58 composite samples (5 eggs/composite) from either the eggshell or egg contents: from 13 poultry farms (65) and 45 retail outlets (225)	January–April 2016	Nsukka and Enugu (Nigeria)	-1.2. -2.4.-2.5. -3.6.-3.4.	43/75 (57.3%) of *S. aureus* isolates		[85]
125 chicken eggs samples were collected from different retail outlets	January–February; and July–August 2019	Pokhara (Nepal)	-1.1. -2.4. -3.5.	-In winter, 34/50 *S. aureus* samples were isolated: 2 (5.88%) were confirmed as MRSA -In summer, 55/75 *S. aureus* samples were isolated: 4 (7.27%) were confirmed as MRSA		[86]
33 raw eggs from street food vendors	September 2015–May 2016	Delhi and Bareilly (India)	-2.3. -3.2.-3.1.	None		[63]
275 eggs from household chickens	November 2016–March 2017	Khyber Pakhtunkhwa (Pakistan)	-1.1. -2.4. -3.4.-3.3.	None		[87]
959 samples representing 8 types of animal-based foods (pork, chicken, beef, duck, lamb, aquatic products, egg, and milk) were collected from randomly selected local markets (including 21 supermarkets and 18 wet markets)	July 2018–August 2019	Shanghai (China)	-1.5. -2.1.1. -3.4.-3.2.	23/165 (13.9%) of *S. aureus* isolates resistant to oxacillin: 2 eggs		[68]
393 poultry egg samples from hatcheries (238), retail shops (94), and households (61)		Ludhiana, Punjab (India)	-1.1. -2.1. -3.2.-3.4.	None		[88]
627 fresh brown eggs from 3 poultry farms and 12 retail markets	August 2013–November 2014	Yangling Shaanxi Province (China)	-1.2. -2.1.1. -3.2.-3.4.	3/627 (0.5%)	-CC45/ST45/t116 (1) -CC5/ST5/t002 (2)	[10]

**Meaning of the numbers used in the detection method column. 1. Pre-enrichment.**: 1.1. Without pre-enrichment; 1.2. Buffered peptone water, BPW; 1.3. Tryptone soy broth (TSB) containing 7.5% NaCl; 1.4. Brain heart infusion (BHI); 1.5. Sodium chloride broth 10%; 1.6. Nutrient broth; 1.7. Saline solution. **2. Solid media**: 2.1. Baird–Parker agar (BP) (2.1.1. BP supplemented with egg yolk tellurite emulsion); 2.2. Blood agar; 2.3. HiChrome MeReSa agar plates with cefoxitin and methicillin supplement; 2.4. Mannitol salt agar (MSA); 2.5. Oxacillin-resistance-screening-agar-base (ORSAB) supplemented with oxacillin (2 mg/L). **3. Identification of MRSA**: 3.1. Cefoxitin (30 μg) and oxacillin (1 μg) disk diffusion susceptibility tests; 3.2. PCR-based amplification of *mec*A gene; 3.3. PCR-based amplification of *mec*A and *mec*C genes; 3.4. Antimicrobial susceptibility testing; 3.5. Cefoxitin disk diffusion method; 3.6. MRSA latex agglutination test (penicillin-binding protein 2a latex agglutination test). Baird–Parker: BP; brain heart infusion: BHI; buffered peptone water: BPW; mannitol salt agar: MSA; tryptone soya broth: TSB.

**Table 3:** Prevalence of MRSA in vegetables, mushrooms, and fruits (in the absence of clarification, the prevalence is considered based on the detection of the *mec*A gene).

Samples	Period	Location	Detection Method	Prevalence	Identification	Reference
120 samples from tomato farms: soils and water, protected houses, packing houses, employees; tomatoes and leaves	2004	Gyeongnam (Korea)	-1.3.2. -2.4.-2.1.1. -3.4.	2/14 (14.3%) of *S. aureus* isolates were resistant to oxacillin: in roller (protected house) and in hydroponic solution -No MRSA in tomatoes and leaves		[89]
616 vegetable samples from supermarkets and conventional markets: lettuce, bean sprouts, green bean sprouts, spinach, a young radish, and Chinese cabbage	January 2003–December 2004	Seoul, Busan, Daejon, and Gwangju (Korea)	-1.3.2. -2.1.1. -3.4.-3.2.	None		[90]
149 strains isolated from samples of food, such as vegetables	2004–2010	Colombia	-1.1. -2.1. -3.4.-3.2.	None		[91]
53 leaf vegetables samples from markets: sprout, lettuce, and perilla	2009–2011	Gyeonggi-do (Korea)	-1.1. -2.1. -3.2.-3.4.	11/53 (20.75%) -Among the 53 isolates, 15 (28.30%) had a positive reaction on the oxacillin disk test, but 4 of these exhibited negative PCR results for amplification of the *mec*A gene		[92]
102 fresh vegetable and fruit samples from local grocery stores: leafy greens, mushrooms, apples, and pear	July 2010–October 2012	Shanghai (China)	-1.1. -3.2.	None		[93]
32 pickled vegetables and 4 vegetables and fruits from different food markets	August–October 2010	Wuhou District of Chengdu City, Sichuan Province (China)	-1.5. -2.1.1. -3.2.-3.4.	2/103 (1.94%) of *S. aureus* isolates from pickled vegetables		[36]
2162 food samples: pasta, rice, fruit, etc. Collected during official monitoring activities	2008	Italy	-1.1. -3.5.-3.4.	None		[94]
339 samples of fruits and vegetables (fresh-cut, whole, and frozen) and sprouts were randomly collected from different supermarkets, local stores, and green markets	2014	Czech Republic	-1.2. -2.2. -3.2.	1/339 (0.3%): frozen fruit (unpacked plums)	ST45/t015 (human origin)	[95]
3000 vegetable salads from supermarket outlets	2015	China	-1.4. -2.1.-2.3. -3.2.-3.4.	1/85 (1.2%) of *S. aureus* isolates of vegetable salads		[84]
50,316 retail market food samples located in 32 provinces	2011	China	-1.4. -2.1.-2.3. -3.2.-3.4.	15 MRSA isolates: 2 in RTE vegetable salads	-ST630 (CC8)/t4549/MLVA Complex MC8 (1 RTE vegetable salad) -ST59 (CC59)/t437/MC621 (1 RTE vegetable salad)	[96]
419 retail vegetable samples from supermarkets, fairs, and farmers’ markets: 110 tomatoes, 128 cucumbers, 84 lettuces, 87 caraway, and 10 other vegetables	July 2011–June 2016	China	-1.3.2. -2.5. -3.4.-3.3.	5/30 (16.7%) of *S. aureus* isolates: 3 from lettuce and 2 from caraway	-ST59/t437 (3/5, 60%) (2 caraway and 1 lettuce) -ST1/t114 (1 lettuce) -ST7/t2874 (1 lettuce)	[97]
Bulk RTE foods: 401 fruit and vegetables	2013–2016	Sichuan (China)	-First: 1.2. -Second: 1.3.3. -2.1.1. -3.2.-3.4.	None		[98]
*S. aureus* strains were isolated from retail foods: 42 isolates from edible mushrooms and 30 isolates from vegetables	July 2011–June 2016	China	-3.5.-3.3.-3.4.	-5/699 (0.7%) edible mushrooms: 7/42 (16.67%) of *S. aureus* isolates -4/419 (1%) vegetables: 5/30 (16.67%) of *S. aureus* isolates	-Edible mushrooms: ·ST59/t437 (7): SCC*mec* IVa (4) and V (3) -Vegetables: ·ST59 (3), ST1 (1) and ST7 (1) ·t437 (3), t114 (1) and t2874 (1) ·SCC*mec* IVa (3), IVd (1) and not detected (1)	[64]
397 samples comprised of 16 different raw leafy vegetables from selected wet markets	May 2015–March 2016	Malaysia	-1.1. -2.1.1. -3.4.-3.2.	9/42 (21.4%) of *S. aureus* isolates		[99]
33 raw vegetables samples from a Moroccan hospital kitchen	May 2015–June 2016	Fez (Morocco)	-1.2. -2.1.1. -3.4.-3.2.	1/7 (14.28%) of *S. aureus* isolates		[100]
143 plant-based products from 10 specialty markets selling imported foods: 88 frozen and 55 dried items	February–May 2017	Saskatoon, Saskatchewan (Canada)	-1.2. -2.4. -3.4.-3.3.	2/7 (28.6%) of *S. aureus* isolates	-ST834 (1) -NT	[4]
350 samples from retail centers: 180 raw vegetable and 170 salad samples	September 2019–January 2020	Chaharmahal Va Bakhtiari province (Iran)	-1.3.1. -2.1.1. -3.1.-3.2.-3.4.	26/45 (57.77%) of *S. aureus* isolates: 8 vegetable and 18 salads		[101]
143 samples of RTE fruits and vegetables were sold at 2 farmers’ markets, 2 supermarkets, and 2 e-commerce stores: 66 samples were fruits (13 fresh-cut honeydew melon, 13 fresh-cut watermelon, 10 cherry tomato, 10 fresh-cut pear, 10 fresh-cut peach, and 10 grape), and 77 were vegetables (37 lettuce, 15 chicory, 6 purple cabbage, 7 cucumber, 5 coriander, and 7 carrot)	June–September 2021	Shanghai (China)	-1.1. -2.1. -3.4.-3.3.	3/47 (6.4%) of *S. aureus* isolates: lettuce, pear, and cherry tomato	SCC*mec*IV/ST7208 (3)	[14]

**Meaning of the numbers used in the detection method column. 1. Pre-enrichment.**: 1.1. Without pre-enrichment; 1.2. Buffered peptone water, BPW; 1.3. Tryptone soy broth (TSB) [TSB containing 10% NaCl and 1% sodium pyruvate; 1.3.2. TSB containing 10% NaCl]; 1.4. Saline solution; 1.5. Brain heart infusion (BHI). **2. Solid media**: 2.1. Baird–Parker agar (BP) (2.1.1. BP supplemented with egg yolk tellurite emulsion); 2.2. Brilliance MRSA Agar (chromogenic selective agar for MRSA); 2.3. Blood agar; 2.4. Mannitol salt agar; 2.5. *S. aureus*-selective CHROM agar plates. **3. Identification of MRSA**: 3.1. Cefoxitin (30 μg) and oxacillin (1 μg) disk diffusion susceptibility tests; 3.2. PCR-based amplification of *mec*A gene; 3.3. PCR-based amplification of *mec*A and *mec*C genes; 3.4. Antimicrobial susceptibility testing; 3.5. Cefoxitin disk diffusion method. Baird–Parker: BP; brain heart infusion: BHI; buffered peptone water: BPW; mannitol salt agar: MSA; not typable: NT; ready-to-eat: RTE; tryptone soya broth: TSB.

**Table 4:** Prevalence of MRSA in different food products (honey, insects, ready-to eat foods; in the absence of clarification, the prevalence is considered based on the detection of the *mec*A gene).

Samples	Period	Location	Detection Method	Prevalence	Identification	Reference
98 samples from the food service environments of elementary schools: drinking water, kitchen utensils, cookers, and cooked foods	November–December 2000	Gyeongnam (Korea)	-1.3.3. -2.4. -3.4.	None		[102]
106 samples of RTE foods from 2 food centers (hospital and student cafeteria), and a local market	A period of 3 months	Songkhla (Thailand)	-Homogenized: 1.2. -1.1. -2.1. -3.4.	22/106 (20.75%) were resistant to oxacillin		[103]
148 presumptive *S. aureus* isolates from 21 food products	2006–2008	Portugal	-1.1. -2.1.2. -3.2.-3.4.	56/148 (38%) of the isolates were resistant to oxacillin		[104]
29 *S. aureus* isolates from different retail processed foods	2003–2006	Korea	-1.1. -3.4.-3.2.	None		[28]
693 food samples associated with foodborne investigations submitted to the Alberta Provincial Laboratory for Public Health, from restaurants, retail facilities, or private homes	January 2007–December 2010	Alberta (Canada)	-1.1. -3.2.	None		[105]
80 RTE salad samples of 4 salads: cheese, fish roe, eggplant, and tzatziki (20 samples of each) 50 samples of 5 basic salad ingredients: feta cheese, mytzithra, mayonnaise, fish roe, and roasted egg pulp (10 samples of each)	Six-month period	Greece	-Two methods: ·1.1. ·1.3.4. -2.1.2. -3.4.	None		[106]
40 cooked meat products and 20 ice creams from markets, a large grand hotel, and farms	April–November 2011	Anhui (China)	-1.1. -3.4.	None		[30]
267 *S. aureus* strains isolated from 244 RTE foods (precooked foods, meat and fish products, cheese, delicatessen salads, sandwiches and canapés, confectionery and bakery products, and various other RTE foods)	August 2007– July 2008	Switzerland	-1.1. -2.11.1. -3.2.-2.9.	2/267 (1%) of *S. aureus* isolates had *mec*A -Two strains had genes involved in methicillin resistance (*mec*A and delta_*mec*R) but were phenotypically methicillin sensitive as determined by streaking them on chromID agar		[107]
120 dumplings, 490 RTE foods, 228 powdered infant formula, and 422 infant rice cereal from supermarkets and farmers markets	July 2008–December 2012	Shaanxi (China)	-First: 1.2. -Second: 1.3.4. -2.1.1. -3.2.-3.4.	-3/490 (0.6%) RTE food samples -3/120 (2.5%) dumpling samples	-ST9/SCC*mec* IVb/t899: 1 dumpling -ST88/II/t10793: 2 dumpling -ST88/V/New *spa* type: 2 dumpling -ST630/SCC*mec* NT/t377: 1 RTE food -ST59/NT/t437: 1 RTE food -ST59/NT/t437: 1 RTE food	[108]
63 samples of prepared foods: 15 chicken, 15 beef, 15 pork, and 18 fish from the kitchens of public hospitals	July 2011–May 2012	Salvador (Brazil)	-First: 1.4. -Second: 1.7. -2.10. -3.7.	6/63 (9.5%): chicken products (1/15, 6.7%), pork products (1/15, 6.7%), and fish products (4/18, 22.2%)		[33]
490 RTE foods, 120 dumplings, and 447 infant rice cereals	July 2008–March 2014	China	-First: 1.2. -Second: 1.3.4. -2.1.1. -3.4.-3.2.	None		[79]
200 food samples sold in a black market at an EU border: 13% others (e.g., eggs, biscuits, spices)	July 2012–February 2013	Galati (Romania)	-1.1. -3.4.-3.3.	None		[35]
100 food samples from the kitchen of a catering firm		Kars (Turkey)	-1.1. -2.1.1. -3.2.	4/100 (4%)		[109]
61 frozen food samples (various quick-frozen dumplings with meat and vegetable fillings) which were stored under the freezing conditions for transportation and sale; 68 processed soybean products (35 fresh tofu, 16 tofu skin, and 17 dried tofu products) from local grocery stores	July 2010–October 2012	Shanghai (China)	-1.1. -3.2.	1/20 (5%) of *S. aureus* isolates in frozen foods -None in processed soybean products	-ST88 (CC88)/*agr* III (1) (HA-MRSA)	[93]
3 rice products, 31 cooked meat products, 15 soybean products, 5 dry cakes, and 3 confections from different food markets	August–October 2010	Wuhou District of Chengdu City, Sichuan Province (China)	-1.5. -2.1.1. -3.2.-3.4.	6/103 (5.83%) of *S. aureus* isolates: 3 cooked meat products, 2 soybean products, and 1 confection		[36]
455 samples of Indian Rojak food items were collected from 35 different Indian Rojak stalls: 321 cooked items with or without re-heating, 99 serving portions of raw vegetables, and 35 serving portions of gravy Additional raw RTE ingredients: 20 raw cucumber, 20 onion, 20 green chili, 40 raw tofu, and 39 fishcake samples from wet markets and supermarkets	September 2011–January 2012	Singapore	-1.1. -2.1. -3.2.-3.7.-3.5.	2/11 of *S. aureus* isolates (18.2%): 1 from onions and 1 from prawn fritters from the same Rojak stall		[81]
120 food samples from doner kebab restaurants, retail markets, butchers, and barns: 20 chicken doner kebabs	July 2012–March 2013	Kırşehir (Turkey)	-First: 1.2. -Second: 1.8. -2.1.1. -3.2.	None		[110]
60 natural untreated and unpasteurized honey samples from different botanical sources purchased in bulk from village open markets		Epirus (Greece)	-1.1. -2.1. -3.4.	8/60 (13.3%) positive *S. aureus*: 35% resistant to oxacillin		[17]
200 samples of raw and RTE Food illegally sold from a market at the eastern EU Border: 13% other food products (spices, dried fruits, jellies, gingerbread, and candies) from the Republic of Moldova, Ukraine, and Bulgaria	July 2012– February 2013	Galati (Romania)	-1.1. -2.1.2. -3.8.	None		[40]
550 RTE foods from retail markets: 119 cooked pork, 153 cooked chicken, 127 cooked duck, 53 cold vegetable dishes in sauce, 52 cold noodles, and 46 fried rice/sushi samples	December 2011–May 2014	China	-1.3.3. -2.5. -3.4.-3.3.	-7/69 of *S. aureus* isolates were identified by cefoxitin disc diffusion test: 2 cold noodles, 2 cooked duck, 1 cooked chicken, 1 fired rice/sushi, and 1 cold vegetables dish in sauce -6 isolates were *mec*A-positive (all except for cold vegetable dishes in sauce)	-ST59/SCC*mec* IVa (2B) (cooked duck) (2/6, 33.3%) (CA-MRSA) -ST338/V (5C2) (cold noodles) (CA-MRSA) -ST3239 (novel ST)/SCC*mec* NT (2/) (cold noodles) -ST9/undescribed SCC*mec* (2C2) (cooked chicken) (LA-MRSA) -ST1/V (5C2) (fired rice/sushi) (CA-MRSA)	[111]
227 *S. aureus* isolates from RTE food and food contact surfaces in a retail environment	2011–2014	Singapore	-3.2.-3.7.-3.5.	5/227 (2.2%) of *S. aureus* isolates: 1 sliced onion, 1 prawn fritters, 1 fried egg, and 2 swabs of food handlers’ gloves	SCC*mec* IV (5) (CA-MRSA): -ST80/t1198 (3) (from sliced onion, prawn fritters, fried egg) -ST6/t304 (2) (from swabs of food handlers’ gloves)	[82]
78 CPS were collected in a routine analysis from several food companies (1454 food samples)	2009	Porto (Portugal)	-1.1. -2.4. -3.2.-3.4.	4/73 (5.5%) of *S. aureus* isolates: 1 from a fermented meat product, 2 from RTE, and 1 from a pastry product -Of the *S. aureus* isolates resistant to oxacillin (5.5%), only one was *mec*A-positive. The other three MRSA strains did not show oxacillin resistance aside from the presence of the *mec*A gene	SCC*mec* IV (1 fermented meat product), V (2 RTE) and NT (1 pastry)	[112]
31 meat barbecue, 82 chicken barbecue, 19 grilled fish, 94 soup, 56 salad, and 119 cooked rice samples from big hospitals	June 2015–June 2016	Isfahan (Iran)	-First: 1.2. -Second: 1.3.1. -2.1.1. -3.1.-3.2.	-12/132 (9.09%) cooked foods with animal origin: 5 meat barbecue and 7 chicken barbecue -11/269 (4.08%) cooked foods without animal origin: 6 soups, 4 salads, and 1 rice -All of the *S. aureus* isolates of meat barbecue, chicken barbecue, soup, and salad samples had complete resistance against methicillin	-The prevalence of bovine-, ovine-, poultry-, and human-based biotypes in the MRSA strains isolated from various types of hospital food samples were 8.10, 8.10, 32.43, and 48.64%, respectively -The biotypes of the 2.70% of MRSA strains were determined as unknown -All of the MRSA strains recovered from soup, salad, and rice samples were related to human-based biotypes	[47]
Sampling of 44 raw and cooked food materials and 65 swab samples of cooking utensils in a hospital kitchen	April 2011–January 2013	Tehran (Iran)	-1.1. -2.4.-2.7.-2.3. -3.4.	-7/44 (16.66%) foods -32/65 (50%) utensils		[113]
910 hospital food samples: 308 meat, 142 poultry, 179 fruit, 455 vegetable, 210 dairy/eggs, 376 bread/grains, 1 nut, and 200 other samples	May 2011–July 2012	St Louis, Missouri (USA)	-1.3.2. -2.3.1.-2.8. -3.6.-3.4.	29/910 (3.2%): meat (7/308, 2%), poultry (2/142, 1%), fruit (2/179, 1%), 14/455 vegetable (3%), 7/210 dairy/eggs (3%), 19/376 bread/grains (5%) and 9/200 other (5%) (veggie burger, sauce, pudding, jelly, 2 gravy, fish, 2 cake)	SCC*mec* II (9/29, 31%), III (2/29, 7%), and IV (18/29, 62%)	[15]
500 different food samples: 137 vegetarian items, 69 non-vegetarian items, 87 dairy products, 72 bakery, and 135 processed products collected from a bakery, market, hospital canteen, and street vendors		Mysuru (India)	-1.2. -2.1.-2.4.2. -3.2.-3.4.-3.6.	152/152 (100%) of *S. aureus* isolates		[114]
Food samples from food processing industries: 105 candy, 50 candy mix, 85 dates, 15 formula milk, 40 lentil, 30 mayonnaise, 100 meat product, 75 paratha, 109 rice, 40 samosa, and 338 spices mix samples	2013–2016	Karachi (Pakistan)	-2.1.1.-2.4.-2.3.-2.5. -3.2.	-10/105 (9.52%) in candies -4/50 (8%) in candy mix -6/85 (7.06%) in dates -3/40 (7.5%) in lentils -3/30 (10%) in mayonnaise -7/100 (7%) in meat products -14/75 (18.67%) in paratha -10/109 (9.17%) in rice -4/40 (10%) in samosa -17/338 (5.03%) in spices mix	-Candies: SCC*mec*A type II (2), III (2), IV (6), *agr* type I (2) and *agr* II (8) -Candy mix: SCC*mec*A II/*agr* II (4) -Dates: SCC*mec*A IV/*agr* II (6) -Lentils: SCC*mec*A IV/*agr* I (1) and *agr* II (2) -Mayonnaise: SCC*mec*A II/*agr* I (3) -Meat products: SCC*mec*A III (3), IV (4), *agr* I (3) and *agr* II (4) -Paratha: SCC*mec*A II (3), IV (11), *agr* I (6) and *agr* II (8) -Rice: SCC*mec*A IV/*agr* I (2) and *agr* II (8) -Samosa: *agr* I/SCC*mec*A II (2) and SCC*mec*A IV (2) -Spices mix: SCC*mec*A IV/*agr* II (17)	[48]
100 cooked meat, 100 cooked chicken, 100 cooked fish, and 70 soup samples from restaurants	February 2015–February 2016	Isfahan (Iran)	-1.2. -2.1.1. -3.2.-3.4.	60/119 (50.42%) of *S. aureus* isolates: 25 cooked meat, 30 cooked chicken, 3 cooked fish, and 2 soup -The prevalence of MRSA strains in all samples was higher in winter seasons than other seasons of the year	-SCC*mec* V (15 cooked meat, 18 cooked chicken, 2 cooked fish, and 2 soup), -IVa (14 cooked meat, 18 cooked chicken, 2 cooked fish, and 2 soup), -IVb (7 cooked meat, 10 cooked chicken, 1 cooked fish, and 1 soup) -II (2 cooked meat and 2 cooked chicken) -I (2 cooked meat and 3 cooked chicken) -III (3 cooked meat, 3 cooked chicken, and 1 cooked fish) -IVc (5 cooked meat, 6 cooked chicken, and 1 cooked fish) -IVd (2 cooked meat and 4 cooked chicken)	[52]
3000 rice- and flour products, 3000 sandwiches, 2000 milk products, 500 condiments, 500 bean products), and 500 fruit desserts from supermarket outlets	2015	China	-1.9. -2.1.-2.3. -3.2.-3.4.	-9/90 (10%) of *S. aureus* isolates of rice- and flour products -5/69 (7.2%) of *S. aureus* isolates of sandwiches -1/14 (7.1%) of *S. aureus* isolates of other foods		[84]
50,316 retail market food samples located in 32 provinces	2011	China	-1.9. -2.1.-2.3. -3.2.-3.4.	15 MRSA isolates: 5 cooked meat and 2 cooked noodles	-ST9 (CC9): t437/MLVA Complex MC621 (2 cooked meat) and t030/MC8 (1 cooked noodles) -ST903 (CC9)/t172/MC621 (1 cooked meat) -ST10 (CC10)/t1244/NMC (1 cooked meat) -ST59 (CC59)/t337/MC621 (1 cooked meat) -ST338 (CC59)/t437/MC621 (1 cooked noodles)	[96]
495 food samples from foodstuffs: 105 cooked dishes with samples of foods prepared with chicken (45, e.g., roasted chicken and chicken stew) and beef (60, e.g., seasoned beef strips, beef stew with vegetables, boeuf rôti, and steak)	November 2014–November 2015	Algeria	-1.1. -2.1.1. -3.3.-3.4.	26/153 (17%): 2 roasted chicken samples and 1 steak -40 *S. aureus* isolates exhibited a methicillin-resistance phenotype -33/153 (21.5%) of *S. aureus* isolates were resistant to cefoxitin	SCC*mec* IV (1 roasted chicken and 1 steak) and V (1 roasted chicken)	[115]
9646 food samples: grain products, meat products, milk products, fishery products, drinks, pastry, fruit products and vegetable products, etc.	2013–2015	Yunnan (China)	-First: 1.2. -Second: 1.3.4. -2.1.1. -3.4.-3.2.	74/251 (29.5%)	-ST1/t1775/SCC*mec* IV (4) -ST5/t002/IV (9) -ST6/t701/IV (27) -ST7/t091: IV (4) and NT (4) -ST59/t437: IV (9) and V (15) -ST88/t10777/IV (2)	[116]
127 *S. aureus* isolates from RTE food samples: 55 from fresh meat, 18 from meat products, 17 from fruits and vegetables, and 37 from cereal products	July 2013–December 2015	Zhengzhou (China)	-3.4.-3.2.	38/127 (29.92%) of *S. aureus* isolates	-t002/*agr*II/SCC*mec* III (6/38) -t701/*agr*I/III (3/38) -t437/*agr*I/IVa (3/38) -SCC*mec* type III (23/38, 60.53%), IVa (4/38, 10.53%), and NT (11/38)	[117]
93 *S. aureus* isolates from different food samples in supermarkets, food markets, and retail stores	2014	Chengdu (China)	-3.4.-3.2.	None detected with the *mec*A gene -14/93 (15.05%) were resistant to oxacillin: 5 cooked meat, 2 pickle, and 1 fresh food samples		[58]
8700 foods from local markets	2007–2015	Sichuan (China)	-1.6. -2.2. -3.2.-3.4.	19/8700 (0.2%): 6 retail food cake, 1 retail food cooked rice, 1 retail food cooked noodle, and 6 retail food cooked pork	-ST965/t062/SCC*mec* III (2 retail food cakes) -ST1/t114/IVb (1 retail food cake) -ST9/IVb: t899 (1 retail food cooked pork) and t1939 (2 retail food cooked pork) -ST59/IVa/t437 (2 retail food cooked pork, 3 retail food cakes, and 1 retail food cooked rice) -ST NT/V/t15995/(1 retail food cooked noodle) -ST338/IVb/t437 (1 retail food cooked pork)	[118]
112 RTE foods and 35 processed raw meat samples from retail vendors		Dhaka (Bangladesh)	-1.1. -2.1. -3.4.-3.2.	-7/112 (6.3%) in RTE foods: burger, sweet, beef kebab, salad, pastry, fuska, and chapati -1/35 (2.9%) in processed raw meat: meat ball	-ST80 (3): t1198 (2, burger and sweet) and t8731 (1, meat ball) -ST6 (2): t304 (1, beef kebab) and t10546 (1, salad) -ST239 (2): t275 (1, pastry) and t037 (1, fuska) -ST361/t315 (1, chapati)	[60]
Bulk RTE foods: 1209 meat products, 200 dairy products, and 350 desserts	2013–2016	Sichuan (China)	-First: 1.2. -Second: 1.3.4. -2.1.1. -3.2.-3.4.	-1/31 (3.23%) of *S. aureus* isolates in meat products -2/8 (25%) of *S. aureus* isolates in desserts -None in dairy products		[98]
120 RTE beef products from some public restaurants and street vendors: kofta, burger, shawarma, and luncheon meat samples (30 of each)		Benha (Egypt)	-3.2.-3.4.	2/8 (25%) of examined isolates -61 *S. aureus* isolates: resistant to oxacillin (70.5%) and methicillin (70.5%)		[119]
107 RTE (lassi, rasmalai, burfi, pedha, curd, rasgulla, salad, chutney, and masala) and 38 cooked food samples (chicken gravy, omelet, cooked fish, boiled egg, and boiled milk) from street food vendors	September 2015–May 2016	Delhi and Bareilly (India)	-2.6. -3.2.-3.1.	None		[63]
*S. aureus* strains were isolated from retail foods: 368 isolates from quick-frozen products (frozen dumplings/steamed stuffed buns and frozen meat), and 148 isolates from RTE food (cold vegetable/noodle dishes in sauce, fried rice/sushi, roast meat, sausage, and ham)	July 2011–June 2016	China	-3.5.-3.3.-3.4.	-9/859 (1.1%) RTE food: 11/148 (7.43%) of *S. aureus* isolates -16/601 (2.7%) quick-frozen food: 20/368 (5.43%) of *S. aureus* isolates	-RTE food: ·ST59 (7), ST9 (1), ST1 (2) and ST338 (1) ·t437 (8), t899 (1), t127 (1) and t085 (1) ·SCC*mec* IVa (7), IVb (1), V (1) and not detected (1) -Quick-frozen food: ·ST59 (5), ST9 (9), ST398 (3), ST630 (1), ST5 (1) and ST10 (1) ·t437 (2), t899 (8), t127 (1), t034 (2), t002 (1), t1751 (1), t441 (2), t377 (1), t4549 (1) and t528 (1) ·SCC*mec* III (1), IVa (5), IVb (9), V (3) and not detected (3)	[64]
150 hot meals, 54 salads, and 24 pastries from Moroccan hospital kitchen	May 2015–June 2016	Fez (Morocco)	-1.2. -2.1.1. -3.4.-3.2.	-2/2 (100%) of *S. aureus* isolates in hot meals -None in salads and pastries		[100]
120 samples (60 samosa and 60 falafel) from licensed food vendors	2015–2016	Hamedan (Iran)	-1.1. -2.1.3. -3.4.-3.2.	10/57 (17.58%) of *S. aureus* isolates: 5 samosa and 5 falafel samples -Only 7 *mec*A-positive isolates were resistant to oxacillin		[120]
83 pastries and 35 sandwiches samples from cafeteria and creameries	2017–2018	Tizi Ouzou (Algeria)	-2.1.1. -3.3.-3.4.	None		[121]
24 commercially processed ready-to-eat products from different vendors: 12 edible worms (*Rhynchophorus phoenicis*) and 12 snails (*Archachatina marginata*)	November–December 2019	Benin City (Nigeria)	-1.1. -2.4.-2.4.1. -3.4.	Resistant to oxacillin: -Edible worm: 9/30 (30%) of *S. aureus* isolates -Snail: 7/22 (31.8%) of *S. aureus* isolates		[18]
357 hospital food samples from 13 hospitals: 321 cooked foods and 36 ready-to-eat salads (36 meat barbecue, 36 chicken barbecue, 36 cooked meat, 36 cooked chicken, 33 fried fish, 36 cooked rice, 36 dill rice, 72 mixed or bean rice (mixed rice cooked with either red meat, poultry, vegetables, and/or beans), and 36 soup and 36 salad samples)	August 2019–January 2020	Mashhad (Iran)	-1.3.3 -2.1.3. -3.1.-3.2.-3.4.	20/87 (22.98%) of *S. aureus* isolates: 1 dill rice, 1 cooked rice, 1 cooked meat, 1 cooked chicken, 2 soup, 2 fried fish, 3 salad, 4 meat barbecue, and 5 chicken barbecue samples		[122]
100 sandwich samples of shawarma poultry meat from fast-food shops		Al-Ahsa (Saudi Arabia)	-1.1. -2.1.1. -3.4.	5/14 (35.7%) of *S. aureus* isolates were found to be positive for MRSA in an antibiogram analysis		[123]
420 RTE food samples, comprising 70 of each food type from vendors: fruit salad, suya (roasted beef), cake, boiled rice, bread, and meat hotpot (cooked meat in tomato sauce)	February–August 2020	Buea (Cameroon)	-1.1. -2.1. -3.2.-3.4.	4/420 (0.95%): 1 fruit salad, 1 bread, and 2 meat hotpot samples		[19]

**Meaning of the numbers used in the detection method column. 1. Pre-enrichment.**: 1.1. Without pre-enrichment; 1.2. BPW; 1.3. TSB (1.3.1. Containing 10% NaCl and 1% sodium pyruvate; 1.3.2. Containing 6.5% NaCl; 1.3.3. Containing 10% NaCl; 1.3.4. Containing 7.5% NaCl); 1.4. MHB Containing 6.5% NaCl; 1.5. BHI broth; 1.6. Enrichment broth containing 10 g/l tryptone, 75 g/l NaCl, 10 g/l mannitol and 2.5 g/l of yeast extract (0.25% yeast extract, 1% mannitol, 1% tryptone, and 7.5% NaCl); 1.7. PHMB Containing ceftizoxime (5 μg/mL) and aztreonam (75 μg/mL); 1.8. GCB; 1.9. Saline solution. **2. Solid media**: 2.1. Baird–Parker agar, BP agar [2.1.1. BP supplemented with egg yolk tellurite emulsion; 2.1.2. BP supplemented with rabbit plasma fibrinogen (BP-RPF); 2.1.3. BP supplemented with egg yolk and potassium tellurite]; 2.2. Selective MRSA agar (BBL CHROM agar MRSA; BD)/MRSA chromogenic agar; 2.3. Blood agar, BA (2.3.1. BA supplemented with sheep’s blood); 2.4. Mannitol salt agar, MSA (2.4.1. MSA with 2 µg/mL oxacillin; 2.4.2. Mannitol salt phenol red agar, MSPRA; 2.5. *S. aureus*-selective CHROM agar plates; 2.6. HiChrome MeReSa agar plates with cefoxitin and methicillin supplement; 2.7. MacConkey agar; 2.8. Spectra MRSA; 2.9. Chrom-ID MRSA agar; 2.10. MRSA-ID; 2.11. Columbia agar, CA (2.11.1. CA supplemented with 5% sheep’s blood). **3. Identification of MRSA**: 3.1. Cefoxitin (30 μg) and oxacillin (1 μg) disk diffusion susceptibility tests; 3.2. PCR-based amplification of *mec*A gene; 3.3. PCR-based amplification of *mec*A and *mec*C genes; 3.4. Antimicrobial susceptibility testing; 3.5. Cefoxitin disk diffusion method; 3.6. MALDI-TOF-MS (mass spectrometry); 3.7. MRSA latex agglutination test (penicillin-binding protein 2a latex agglutination test); 3.8. *S. aureus spa* typing. Baird–Parker: BP; brain heart infusion: BHI; buffered peptone water: BPW; coagulase-positive *Staphylococcus*: CPS; Giolitti–Cantoni broth: GCB; mannitol salt agar: MSA; Mueller–Hinton broth: MHB; not typable: NT; phenol red mannitol broth: PHMB; ready-to-eat: RTE; tryptone soya broth: TSB.

**Table 5:** Prevalence of MRSA in drinking water (in the absence of clarification, the prevalence is considered based on the detection of the *mec*A gene).

Samples	Period	Location	Detection Method	Prevalence	Identification	Reference
98 samples from the food service environments of elementary schools: drinking water, kitchen utensils, cookers, and cooked foods	November–December 2000	Gyeongnam (Korea)	-1.2. -2.4. -3.4.	1/5 (20%) of drinking water samples were resistant to oxacillin		[102]
2 sampling campaigns were conducted in order to collect 18, 26, and 26 water samples from River Nile, treated raw water, and groundwater, respectively	October 2011–March 2012	Sohag (Egypt)	-3.2.-3.4.	None		[124]
16 wastewater samples (8 influent, 8 effluent) of 6 urban wastewater treatment plants (UWTP)	December 2012–February 2013	La Rioja (Spain)	-Two methods: ·1.1. ·1.3. -2.4.-2.5. -3.4.-3.3.	Only ST398 *S. aureus*: obtained from an effluent water sample	-ST398/t011	[125]
50 drinking water specimens from drinking water supplies		Al-Anbar Province (Iraq)	-1.1. -2.6.-2.3.-2.4. -3.4.	12/50 (24%)		[126]
552 water samples were collected monthly from 4 public parks: 468 drinking water fountains and 84 biofilm from mist makers	March 2017–March 2018	São Paulo city	-2.1. -3.1.-3.2.	The *mec*A gene was only detected in samples from drinking water fountains. Out of 30 isolates, 11 carried the *mec*A gene (36.7%)		[23]
48 grab samples from a drinking water reservoir system	August 2018–June 2019	Germany	-1.1. -2.2. -3.4.	None		[127]
50 water samples from domestic storage tanks	Winter of 2019	Sidon (Lebanon)	-2.4. -3.1.-3.2.	Of the detected *S. aureus* samples, 21% were resistant to the cefoxitin agent: 79.3% of the samples were *mec*A-negative and 20.7% were *mec*A-positive		[21]

**Meaning of the numbers used in the detection method column. 1. Pre-enrichment.**: 1.1. Without pre-enrichment; 1.2. Tryptone soy broth (TSB) containing 10% NaCl; 1.3. Brain heart infusion broth (BHI) containing 6.5% NaCl. **2. Solid media**: 2.1. Baird–Parker agar (BP); 2.2. Selective MRSA agar (BBL CHROM agar MRSA; BD)/MRSA chromogenic agar; 2.3. Blood agar; 2.4. Mannitol salt agar, MSA; 2.5. Oxacillin-resistance-screening-agar-base (ORSAB) supplemented with oxacillin (2 mg/L); 2.6. MacConkey agar. **3. Identification of MRSA**: 3.1. Cefoxitin (30 μg) and oxacillin (1 μg) disk diffusion susceptibility tests; 3.2. PCR-based amplification of *mec*A gene; 3.3. PCR-based amplification of *mec*A and *mec*C genes; 3.4. Antimicrobial susceptibility testing. Baird–Parker: BP; Brain heart infusion: BHI; mannitol salt agar: MSA; tryptone soya broth: TSB.

The prevalence of MRSA varied between the different food groups evaluated and within the groups, depending on the techniques used and the number of samples, which does not allow for consistent comparisons between research works. The percentage of samples contaminated with MRSA ranged between 0.0% and 100% (average 11.7 ± 20.3%) for fish and shellfish, between 0.0% and 11.0% (average 1.2 ± 3.5%) for eggs and egg products, between 0.0% and 20.8% (average 2.5% ± 6.8%) for foods of vegetable origin, between 0.6% and 29.5% (average 28.2 ± 30.3%) for the group of other foods, and between 0.0% and 36.7% (average 17.0 ± 14.0%) for drinking water. The percentage of isolates identified as MRSA within *S. aureus* isolates ranged from 0.4% to 53.8% (average 19.3 ± 13.5%) for fish and shellfish, from 0.44% to 66.7% (average 24.9 ± 24.9%) for egg and egg products, from 1.2% to 57.8% (average 18.1 ± 17.4%) for foods of vegetable origin, and from 1.0% to 100% (average 28.2 ± 30.25%) for other foods. Furthermore, of the 147 articles evaluated, the most used technique to confirm the presence of MRSA was amplification of the *mec*A gene by PCR in 102 of the articles (69.4%), while only 23 of the articles (15.6%) describe the amplification of the *mec*A and *mec*C genes.

## 4. Discussion

The presence of MRSA in foods has a double interest for public health. On the one hand, some MRSA strains produce enterotoxins responsible for food poisoning when favorable conditions for growth and production of toxins exist in the food. There are no differences in the severity of poisoning caused by MSSA and MRSA, because the strain’s antibiotic resistance does not influence toxin production [128]. On the other hand, it has been suggested that MRSA strains present in food can cause infections in the consumer, although it is not yet clear whether this microorganism can act as a foodborne pathogen [129]. For all these reasons, it is advisable to expand knowledge about the epidemiology of MRSA, including its prevalence in different food groups. To the best of our knowledge, this is the first review carried out on the prevalence and types of MRSA in several types of foodstuffs and drinking water. 

Anthropogenic activities, such as wastewater from hospitals, livestock farms, crop fields, and wastewater treatment plants, contribute to the spread of antimicrobial-resistant pathogenic bacteria in the environment. Surface water sources, including river waters, act as critical points for the discharge of wastewater, pollutants, antibiotic-resistant bacteria (ARB), and antibiotic-resistant genes (ARG). These environmental factors, along with others, facilitate the dissemination and survival of ARB, in addition to promoting the exchange of ARG [130]. Contamination of food with MRSA depends on its hygienic state, and can come from an infected person, poor hygiene practices, or unhygienic utensils. People are frequently asymptomatic carriers and are responsible for the continued spread of MRSA in food, from handlers to food and vice versa [8]. The current findings emphasize the need for good hygiene practices (GHP) at multiple processing steps, starting with transportation and retail outlets, to limit the risk of transmission of *S. aureus* and MRSA from food products to humans [131]. 

Multidrug-resistant bacteria are a serious problem that affects public health and the global economy through increased morbidity and mortality rates, longer duration of illness, and high hospital costs [132]. Diseases caused by antibiotic-resistant foodborne pathogens are among the potential bacterial diseases of public health importance identified in fish. Eating raw or undercooked fish can expose consumers to these bacteria and lead to colonization and infection [6]. Furthermore, prolonged use of antibiotics and their misuse in aquaculture has been associated with environmental pollution, resulting in the development of resistant bacteria in the aquatic environment [133]. Differences in the prevalence of MRSA in fish in different studies may be attributed to different sampling times, sample size, geographical location, fish species, post-harvest contamination, and sanitary conditions during transportation, storage, and processing [134].

Different pathogenic microorganisms, including *Salmonella* spp., *Escherichia coli*, and *S. aureus*, cause foodborne illnesses by egg consumption. *S. aureus* is not as important a bacteria as other pathogens in fresh eggs. Some of the suspected sources of egg contamination are egg boxes/shelves, feces, egg packaging and storage equipment, clothing, workers’ hands, dust, and the environment. In contact with dirty surfaces, such as feces, bacteria can contaminate the shell in a short time and penetrate the interior of the egg. People and egg trays can be a source of contamination in grocery stores and retailers [135]. 

Fruits and vegetables are often processed through frequent manual contact, indicating significant potential for *S. aureus* contamination from both human and environmental sources [14]. Bacteria present on cutting tools, cutting boards, or handlers could be transferred to the surface of freshly cut fruit, which, combined with inadequate refrigeration or prolonged storage, creates an environment conducive to bacterial growth [136]. Ready-to-eat (RTE) foods pose unique food safety issues because they are consumed without additional processing, making them susceptible to transmitting pathogens, such as *S. aureus* [137]. Most types of foods, including RTE foods, support the growth of staphylococci and are ideal for the production of enterotoxins. High levels of bacterial contamination of ready-to-eat fruits could be due to several factors, such as washing fruits with poor quality water, cross contamination with other fruits washed in the same water, use of dirty trays or dirty processing utensils, such as knives and cutting tables, failure to wash hands thoroughly, and air contamination [19].

Methods to isolate MRSA from foods should include the enrichment of samples. This increases the frequency of MRSA detection. Since staphylococci are relatively tolerant to high salt concentrations, culture broths with 6.5% or 7.5% NaCl are commonly used in MRSA isolation protocols from different samples, which limits the growth of the contaminating microbiota [25]. Strains with *mec*C are sometimes confused with methicillin-susceptible *S. aureus* (MSSA), raising important implications for MRSA monitoring. To avoid these problems, laboratories should incorporate universal *mec* gene primers for PCR detection or add *mec*C-specific primers to differentiate between *mec*A and *mec*C MRSA. When *S. aureus* isolates are negative for *mec*A and *mec*C in the detection of MRSA, but show resistance to methicillin, the presence of the plasmid carrying the *mec*B gene should be investigated [24]. 

Molecular characterization of *S. aureus* is essential to trace the origin and spread of infection and contamination, as well as to gain a deeper epidemiological understanding of foods. Both multilocus sequence typing (MLST) and staphylococcal protein A typing (*spa*) have been shown to be effective in characterizing and identifying genetic relationships between MRSA isolates [14]. MLST has proven to be very useful for macroepidemiological and evolutionary studies. *Spa* typing shows excellent discriminatory power and shares with MLST the advantages of unambiguous typing results that can be compared across laboratories and over time. *Spa* typing is easier and less expensive to perform than MLST [138]. Data on the characterization of *S. aureus* have primarily focused on clinical settings, foods of animal origin, and dairy products, and limited information is available on fruits and vegetables [14]. This highlights the need for more studies focused on these types of foods.

Another promising tool for MRSA analysis is whole-genome sequencing (WGS). WGS of bacterial pathogens has demonstrated its potential for epidemiological surveillance, outbreak detection, and infection control. This technology aids in the tracking of AMR genes and mobile genetic elements, providing the necessary information for the application of quantitative risk assessments and enabling the identification of AMR hot spots and transmission routes through the food chain [139]. WGS is currently a powerful, very affordable and rapid tool for microbial typing, which also achieves much higher resolution than traditional typing methods, such as multilocus variable number tandem repeat analysis (MLVA), pulsed field gel electrophoresis (PFGE), random amplified polymorphic DNA (RAPD), and multilocus sequence typing (MLST) [139]. WGS is a promising alternative to culture methods for resistance prediction in *S. aureus*, as it is as sensitive and specific as routine antimicrobial susceptibility testing methods [140]. The data obtained by WGS facilitate studies on the epidemiology of foodborne *S. aureus*, as well as on the plasticity of its genome in terms of the acquisition of various genetic elements related to host adaptation, antimicrobial resistance, and virulence. Furthermore, the results obtained allow the development of preventive measures against human infections caused by this important pathogen in community settings [141]. WGS provides information at the genotypic level, and full gene detection allows for expanded knowledge of *S. aureus* and the potential of isolates to produce staphylococcal enterotoxins. In addition, WGS can more accurately identify the strains causing an outbreak of staphylococcal food poisoning and, thus, accelerate its management [142].

## 5. Conclusions

The ability of MRSA strains to cause staphylococcal poisoning and their controversial role in foodborne infections, together with the high prevalence of this microorganism in different foodstuffs (up to 100% of the samples were contaminated in some of the consulted works) highlight the importance of establishing the necessary measures to reduce the presence of MRSA in food and drinking water. The methods utilized in studying MRSA by different authors varied considerably. For this reason, and with an eye to permitting more meaningful comparisons between one investigation and another, it would be advisable to develop a standardized protocol for the study of this microorganism in food and water. In most of the research works consulted, only the *mec*A gene was investigated in determining the presence of MRSA in foodstuffs. However, in order to avoid false negatives, it is crucial to also to check for the *mec*B and *mec*C genes.

## Data Availability

No new data were created or analyzed in this study. Data sharing is not applicable to this article.
